# The relationship between radiation doses to coronary arteries and location of coronary stenosis requiring intervention in breast cancer survivors

**DOI:** 10.1186/s13014-019-1242-z

**Published:** 2019-03-07

**Authors:** Anna-Karin Wennstig, Hans Garmo, Ulf Isacsson, Giovanna Gagliardi, Niina Rintelä, Bo Lagerqvist, Lars Holmberg, Carl Blomqvist, Malin Sund, Greger Nilsson

**Affiliations:** 1 Department of Surgical and Perioperative Science, Surgery, Umeå University, SE-901 85 Umeå, Sweden; 2 Department of Oncology, Sundsvall Hospital, SE-85186 Sundsvall, Sweden; 3 Translational Oncology & Urology Research (TOUR), School of Cancer and Pharmaceutical Sciences, King’s College London, London, UK; 4 Regional Cancer Centre, SE-75185 Uppsala, Sweden; 5 Department of Immunology, Genetics and Pathology, Section of Medical Radiation Science, Uppsala University, University Hospital, SE-751 85 Uppsala, Sweden; 6 Department of Medical Radiation Physics and Nuclear Medicine, Karolinska University Hospital, SE-17176 Stockholm, Sweden; 7 Department of Medical Sciences, Uppsala University, SE-75185 Uppsala, Sweden; 8 Department of Surgical Sciences, Uppsala University, SE-75185 Uppsala, Sweden; 9 Department of Oncology, Örebro University, University Hospital, SE-701 82 Örebro, Sweden; 10 Department of Immunology, Genetics and Pathology, Section of Experimental and Clinical Oncology, Uppsala University, University Hospital, SE-751 85 Uppsala, Sweden; 11 Department of Oncology, Gävle Hospital, SE-801 07 Gävle, Sweden; 12 Department of Oncology, Visby Hospital, SE-621 84 Visby, Sweden

**Keywords:** Breast cancer, Radiotherapy, Radiation doses, 3DCRT, Coronary stenosis, Left anterior descending artery

## Abstract

**Background:**

To assess the relationship between radiation doses to the coronary arteries (CAs) and location of a coronary stenosis that required intervention after three-dimensional conformal radiotherapy (3DCRT) for breast cancer (BC).

**Methods:**

The study population consisted of 182 women treated for BC in Sweden between 1992 and 2012. All women received 3DCRT and subsequently underwent coronary angiography due to a suspected coronary event. CA segments were delineated in the patient’s original planning-CT and radiation doses were recalculated based on the dose distribution of the original radiotherapy (RT) plan. The location of the CA stenosis that required intervention was identified from the Swedish Coronary Angiography and Angioplasty Registry (SCAAR). Logistic regression analysis was used to assess the relationship between CA radiation doses and risk of a later coronary intervention at this specific location.

**Results:**

The odds ratio (OR) varied by radiation dose to the mid left anterior descending artery (LAD) (*p* = 0.005). Women receiving mean doses of 1–5 Gray (Gy) to the mid LAD had an adjusted OR of 0.90 (95% CI 0.47–1.74) for a later coronary intervention compared to women receiving mean doses of 0–1 Gy to the mid LAD. In women receiving mean doses of 5–20 Gy to the mid LAD, an adjusted OR of 1.24 (95% CI 0.52–2.95) was observed, which increased to an OR of 5.23 (95% CI 2.01–13.6) for mean doses over 20 Gy, when compared to women receiving mean doses of 0–1 Gy to the mid LAD.

**Conclusions:**

In women receiving conventional 3DCRT for BC between 1992 and 2012, radiation doses to the LAD remained high and were associated with an increased requirement of coronary intervention in mid LAD. The results support that the LAD radiation dose should be considered in RT treatment planning and that the dose should be kept as low as possible. Minimising the dose to LAD is expected to diminish the risk of later radiation-induced stenosis.

**Electronic supplementary material:**

The online version of this article (10.1186/s13014-019-1242-z) contains supplementary material, which is available to authorized users.

## Background

Adjuvant radiotherapy (RT) in early breast cancer (BC) significantly reduces local recurrences and death in BC and is considered as a part of standard treatment [1, 2]. However, due to incidental radiation exposure to the heart, RT has been associated with increased risk of ischemic heart disease (IHD) [3–6]. Dosimetry studies have shown that the left anterior descending artery (LAD) receives the highest radiation doses in RT for left-sided BC [7, 8] and a higher incidence of LAD stenosis has been reported after RT of left-sided compared to right-sided BC [9, 10]. Radiation volumes and doses to the heart have changed with the development of new techniques, although doses to the anterior part of the heart may still be high [7, 11, 12]. RT techniques using active breathing control (ABC) have shown to further reduce the radiation doses to the heart and LAD [13, 14].

There are uncertainties concerning the dose-response relationship between absorbed dose to the whole heart and the coronary arteries (CAs), and subsequent coronary events [15]. A large population-based study by Darby et al. showed that the rate of major coronary events increased by 7.4% for each increase of 1 Gray (Gy) of the median heart dose (MHD), but the estimated dose to the LAD did not improve the prediction of coronary events [4]. The patients were treated before the era of 3D conformal RT (3DCRT) and radiation doses to the heart and to the LAD were estimated from models [4]. An association between the MHD and coronary events was recently verified in patients receiving contemporary 3DCRT, with an even stronger association between the volume of the left ventricle receiving more than 5 Gy (LV-V5) and subsequent coronary events [16]. Taylor et al. have shown, using simulated heart doses and more contemporary RT, an excess of risk rate of 4.1% per Gy of the MHD [17]. A recent study from the same group showed a significant correlation between simulated doses to LAD and later stenosis [18]. The aim of the present study was to examine whether there is a relationship between radiation dose to the CAs and a later coronary stenosis at this location that requires a coronary intervention. All patients received contemporary 3DCRT, which enables patient-specific information on radiation doses to the CAs. The main focus was on the high-dose region in the LAD.

## Methods

### Patients

The study was designed as a case-only study of women irradiated for BC and who subsequently underwent coronary angiography. To identify the study base, women with BC who had received RT between 1992 and 2012 were selected from the regional breast cancer registries in three of Sweden’s six health care regions (Uppsala-Örebro, Stockholm, and the Northern region). The study base was then linked to the Swedish Coronary Angiography and Angioplasty Registry (SCAAR), a part of the nationwide Swedish cardiac register SWEDEHEART [19], to identify BC patients with a coronary event after RT. Out of these patients, only women receiving 3DCRT and for whom the original RT plan was available, were included in the analysis. Exclusion criteria were bilateral BC and coronary events registered before RT. Clinicopathological information was obtained from the breast cancer registries and targets, fractionation, and total radiation dose were retrieved from the RT charts. Type of coronary investigation (only angiography or intervention), and in case of intervention, location and type of coronary intervention, body mass index (BMI), and smoking status was obtained from SCAAR. The ethical committee of Northern Sweden approved the study.

### Radiotherapy

All information concerning the RT was obtained from the patients’ individual RT charts. The treatment targets included the breast tissue alone; the breast tissue and locoregional lymph nodes (including the axillar, infra-and supraclavicular lymph nodes); breast tissue, locoregional lymph nodes, and internal mammary chain (IMC); the chest wall alone; the chest wall and locoregional lymph nodes; the chest wall, locoregional lymph nodes, and IMC. A number of different fractionation schedules were used, including hypofractionated schedules, conventionally fractionated schedules, and for a number of patients, additional boost fields to the tumour bed. Details regarding radiation targets and fractionation are shown in Table 1. All included patients were treated with conventional treatment techniques, using tangential photon beams or a combination of photon and electron beams. None of the patients received RT with ABC techniques.

**Table 1:** Patients characteristics

	Right-sided BC (*N* = 81)	Left-sided BC (*N* = 101)	All (*N* = 182)	*P*-value
Year of cancer diagnosis, *N* (%)				
1995–2001	21 (25.9)	31 (30.7)	52 (28.6)	0.76
2002–2008	37 (45.7)	41 (40.6)	78 (42.9)	
2009–2012	23 (28.4)	29 (28.7)	52 (28.6)	
Age at cancer diagnosis, *N* (%)				
40–49 yrs	4 (4.9)	6 (5.9)	10 (5.5)	0.90
50–59 yrs	19 (23.5)	27 (26.7)	46 (25.3)	
60–69 yrs	41 (50.6)	44 (43.6)	85 (46.7)	
70–79 yrs	15 (18.5)	22 (21.8)	37 (20.3)	
80–83 yrs	2 (2.5)	2 (2.0)	4 (2.2)	
Age at diagnosis, median (min-max)	64 (40–84)	64 (44–82)	64 (40–84)	
Type of surgery, *N* (%)				
Breast conserving surgery	67 (83.8)	88 (87.0)	155 (85.2)	0.79
Mastectomy	13 (16.2)	11 (12.0)	24 (13.1)	
No surgery	0 (0.0)	1 (1.0)	1 (0.5)	
Missing data	1 (1.2)	1 (1.0)	2 (1.1)	
Endocrine therapy, *N* (%)				
Endocrine therapy	44 (54.3)	52 (51.5)	96 (52.7)	0.77
No endocrine therapy	37 (45.7)	49 (48.5)	86 (47.3)	
Chemotherapy, *N* (%)				
Chemotherapy	23 (28.4)	18 (17.8)	41 (22.5)	0.11
No chemotherapy	58 (71.6)	83 (82.2)	141 (77.5)	
CT-scan slice thickness, *N* (%)				
< 5 mm	19 (23.5)	20 (19.8)	39 (21.4)	0.92
5 mm	21 (25.9)	28 (27.7)	49 (26.9)	
6–9 mm	14 (17.3)	20 (19.8)	34 (18.7)	
10 mm	25 (30.9)	33 (32.7)	60 (32.9)	
15–16 mm	2 (2.5)	0 (0)	2 (1.1)	
Treatment planning system, *N* (%)				
TMS	47 (58.0)	59 (58.4)	106 (58.2)	1.0
Oncentra	8 (9.9)	9 (8.9)	17 (9.3)	
Eclipse	26 (32.1)	33 (32.7)	59 (32.4)	
Algorithm, *N* (%)				
AAA	47 (58.0)	60 (59.4)	107 (58.8)	0.20
CC	21 (25.9)	33 (32.7)	54 (29.7)	
PB	13 (16.0)	8 (7.9)	21 (11.5)	
Target, *N* (%)				
Breast	60 (74.1)	80 (79.2)	140 (76.9)	0.82
Breast + regional LN	8 (9.9)	9 (8.9)	17 (9.3)	
Breast + regional LN + IMC	0 (0.0)	1 (1.0)	1 (0.5)	
Chest wall	1 (1.2)	1 (1.0)	2 (1.1)	
Chest wall + regional LN	10 (12.3)	7 (6.9)	17 (9.3)	
Chest wall + regional LN + IMC	2 (2.5)	3 (3.0)	5 (2.7)	
Fractionation, *N* (%)				
2.66 Gy × 16	16 (19.8)	3 (3.0)	19 (10.4)	0.001
2.67 Gy × 15	2 (2.5)	1 (1.0)	3 (1.6)	
2 Gy x (22–24)	3 (3.7)	2 (2.0)	5 (2.7)	
2 Gy x (22–24) + boost 2 Gy × 9	0 (0.0)	1 (1.0)	1 (0.5)	
2 Gy x (26–28)	10 (12.3)	8 (7.9)	18 (9.9)	
2 Gy × 25	47 (58.0)	83 (82.2)	130 (71.4)	
2 Gy × 25 + boost 2 Gy × 5	2 (2.5)	1 (1.0)	3 (1.6)	
2 Gy × 25 + boost 2 Gy × 8	1 (1.2)	2 (2.0)	3 (1.6)	
Time from RT to SCAAR registration, *N* (%)				
< 2 yrs	22 (27.2)	29 (28.7)	51 (28.0)	0.85
2–4 yrs	15 (18.5)	22 (21.8)	37 (20.3)	
4–8 yrs	21 (25.9)	27 (26.7)	48 (26.4)	
8+ yrs	23 (28.4)	23 (22.8)	46 (25.3)	
Time in years from RT to SCAAR registration, median (min-max)	4.9 (0.1–15.6)	3.9 (0.1–14.0)	5.0 (0.1–15.6)	

Patients characteristics by laterality of the tumor. *BC* Breast cancer, *yrs* Years, *min* Minimum, *max* Maximum, *CT* Computed tomography, *AAA* Analytical anisotropic algorithm, *CC* Collapsed cone, *PB* Pencil beam, *regional LN* Regional lymph nodes i.e.; axillary and supraclavicular lymph nodes, *IMC* Internal mammary chain, *Gy* Gray, *RT* Radiotherapy, and *SCAAR* Swedish coronary angiography and angioplasty registry

### Delineation of organs at risk

**Fig. 1:**
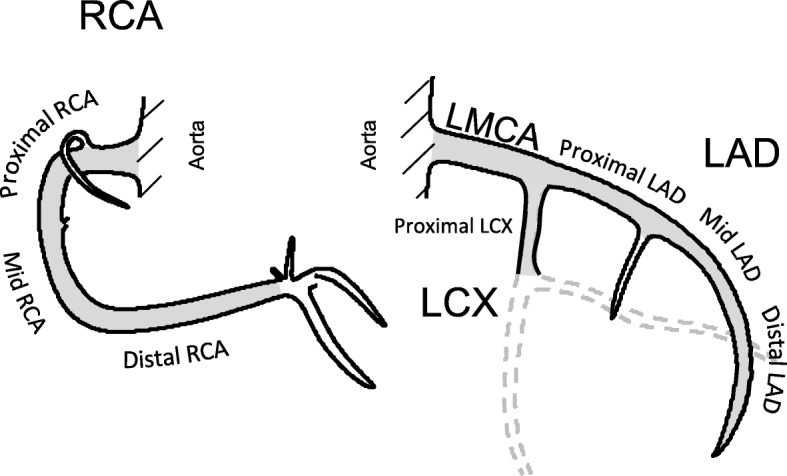
The coronay arteries. The coronary arteries; the right coronary artery (RCA), the left main coronary artery (LMCA), the left anterior descending artery (LAD), and the left circumflex artery (LCX)

All planning-CTs were performed without intravenous contrast enhancement. The heart atlas by Feng et al. [20]. was used as a guideline for the delineation of the CAs and the heart. The left main coronary artery (LMCA), the LAD, the proximal left circumflex artery (LCX), and the right coronary artery (RCA) were delineated. The arteries were divided into segments to localise the stenosis, according to the American Heart Association guidelines [21], which is consistent with the registration in SCAAR (Fig. 1). In the majority of the CT slices, the CAs were visible, but occasionally anatomical landmarks were followed and the interpolation function in the treatment planning system (TPS) used. Contouring was performed by hand with either a minimum brush of 4 mm or with the point-to-point tool, and was adjusted to encompass the arteries with a diameter of about 4 to 6 mm. No extra margin was added. The patients were contoured by one of the authors and the procedure was performed blinded regarding the location of the stenosis. The slice thickness of the CT scans varied from 2 to 16 mm and the majority of the patients (78.6%) had CT scans with slice thickness between 5 and 10 mm.

### Reconstruction of delivered dose to cardiac structures

The original planning-CT and delivered treatment plan were retrieved for each patient in the local TPS of the department. The TPS used were TMS® (Helax AB, Sweden), Oncentra® (Elekta, Sweden), and Eclipse® (Varian Medical Systems Inc., USA). The CA segments and the whole heart were delineated in each patient’s original planning-CT and radiation doses to these structures for each individual patient were recalculated using the original dose distributions. The treatment plans were recalculated whenever possible, with the original accelerator and beam model. If the beam data was not available, the plans were recalculated with newer accelerators and beam models similar to the original, in order to receive a high agreement between the recalculated dose distributions to the CAs and the original dose given. For patients receiving boost fields, the boost dose was included in the dose calculations. Depending on the local practice of the departments, three different algorithms were used: The Pencil Beam (PB) (TMS® and Oncentra®), the Collapsed Cone (CC) (Oncentra®), or the Analytical Anisotropic Algorithm (AAA) (Eclipse®). In order to estimate the difference in dose estimation to the CAs between the PB and the CC algorithms, the RT plans of two patients in the present study with right-sided and left-sided RT respectively, were recalculated with both algorithms. From the recalculated treatment plans, dose-volume histograms (DVH) were generated for the whole heart and the delineated CAs. Mean, median, and maximum radiation doses were determined for the heart and for the CAs in each patient’s individual treatment plan. The maximum dose was defined as the dose to the single calculation point receiving the highest dose.

### Statistical methods

The distributions of radiation dose exposures in all women were summarised in proportions and in descriptive statistics using minimum, first quartile (Q1), median, third quartile (Q3), and maximum. In coronary segment specific analyses, the relation between radiation dose and coronary intervention (yes/no) was compared using logistic regression. To account for the elapsed time since the RT, this time was defined segment-wise. In case of an intervention, the elapsed treatment time was calculated from RT start to date of intervention as recorded in SCAAR. If no intervention was recorded, the elapsed treatment time was calculated from start of RT to last day of follow-up (23rd August 2016) or date of death, whichever came first. In this case-only study, the elapsed treatment time cannot be used directly in the logistic regression as it relates to the outcome in a censoring like manner. Therefore, an actuarial approach to the issue was applied and the elapsed treatment time was split in four-week periods. The data was then transferred to long format based on the actuarial four-week periods, and logistic regression analyses were performed.

Odds ratios (OR) of coronary intervention by each coronary segment were estimated. The ORs correspond to the relative risk of receiving segment-specific interventions comparing different doses. Models using dose as a continuous variable (trend test) but also models where the dose was discretised into categories were performed. For the proximal, mid, and distal RCA, the LMCA, the proximal LAD, and the LCX, cut-offs for the lowest category were chosen as the integer value closes to the median, which represented 1 Gy. In the mid and distal LAD, the cut-offs were chosen according to the 40th and 80th percentile, which represented 1 and 20 Gy, respectively, and for the mid LAD the 70th percentile, representing 5 Gy, was also used as cut-off. Doses in the lowest range were defined as references. The model was adjusted for age (continuous) and year of BC diagnosis (continuous), year of registration in SCAAR (continuous), endocrine therapy (yes/no), chemotherapy (yes/no), BMI (five categories), and smoking status (three categories). The same adjustments were included in models used to assess the risk in relation to time and the combination of time and dose.

All analyses were performed using the statistical software R [22].

## Results

### Patient characteristics

A total of 872 women with a BC diagnosis and a match in SCAAR were identified (Additional file 1: Figure S1). Out of these, 602 patients received adjuvant RT, and their original treatment plans were retrieved. A total of 312 patients were excluded due to lack of a full 3D representation of the RT plan or due to technical issues with reading back RT plans from archives. Furthermore, 85 patients were excluded due to missing RT plans (registration errors or RT received in other health care regions). Finally, eight patients were excluded due to bilateral BC, and 15 patients due to a SCAAR entry before RT. This left a total of 182 patients in the final study base, with 101 patients treated for left-sided BC and 81 for right-sided BC.

Patient characteristics are shown in Table 1. One hundred and fifty-five (85.2%) patients had breast conserving surgery (BCS) performed and 140 (76.9%) of these received RT to the breast without lymph node irradiation. Ninety-six (52.7%) and 41 (22.5%) of the patients received endocrine therapy and chemotherapy, respectively. The RT was planned in the TMS system for the majority of the patients. Doses were recalculated for 107 patients (58.5%) using the AAA, 54 (29.7%) with the CC, and 21 (11.5%) with the PB. Only subtle differences were observed between the PB and the CC algorithms, when the RT plans were recalculated with both algorithms, for one patient with right-sided and left-sided RT, respectively (Additional file 1: Table S1).

Information from the SCAAR, tumour characteristics, mode of cancer detection, and health care region is presented in Additional file 1: Table S2. Percutaneous coronary intervention (PCI) was performed in 172 of the patients and coronary artery by-pass grafting (CABG) in four of the patients. Ten patients underwent coronary angiography without any coronary intervention. Myocardial infarction was the most common indication for intervention and stenosis in one artery branch the most common finding on the coronary angiography. The number and type of coronary interventions are shown in Table 2, presented by each coronary segment separately.

**Table 2:** Coronary intervention presented by each coronary segment separately

	Proximal RCA	Mid RCA	Distal RCA	LMCA	Proximal LAD	Mid LAD	Distal LAD	Proximal LCX
Stent	35	41	14	4	41	50	6	22
Intervention without stent	0	1	1	0	2	0	1	3
Coronary artery by-pass grafting	1	2	1	1	1	2	0	3
Coronary segment not intervened	146	138	166	177	138	130	175	154

Coronary intervention presented by each coronary segment separately. *RCA* Right coronary artery, *LMCA* Left main coronary artery, *LAD* Left anterior descending artery, and *LCX* Left circumflex artery

### Radiation doses

**Fig. 2:**
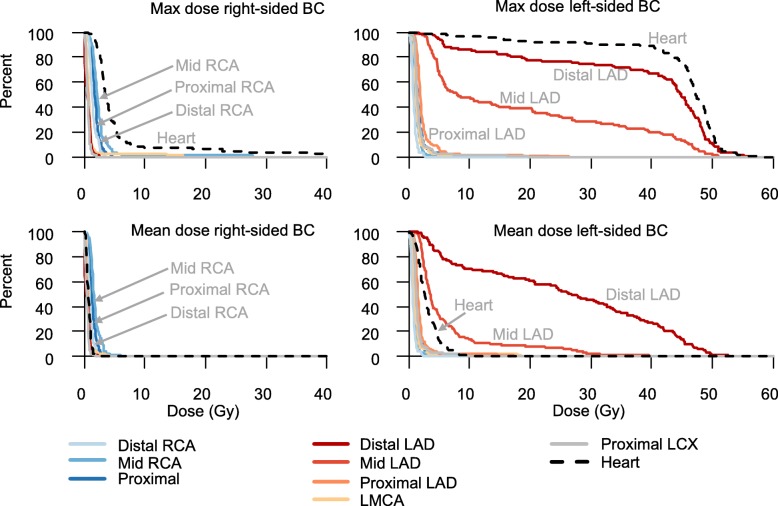
Distribution of coronary radiation doses. Percent of women (Y-axis), receiving at least the radiation dose to the heart and to eight coronary segments as displayed on the X-axis. Results presented by breast cancer laterality, and maximum and mean doses. Breast cancer (BC), Gray (Gy), right coronary artery (RCA), left main coronary artery (LMCA), left anterior descending artery (LAD), and left circumflex artery (LCX)

In right-sided BC RT, the highest radiation doses were seen in mid RCA with a median mean dose of 1.6 Gy and a median maximum dose of 2.2 Gy. The median mean heart dose was 0.6 Gy. In left-sided BC RT, the highest doses were found in the mid and distal LAD, with median mean doses of 3.6 Gy and 26.7 Gy, and median maximum doses of 7.9 Gy and 44.8 Gy, respectively (Fig. 2 and Additional file 1: Table S3). The median mean doses to the whole LAD and the heart was 10.6 Gy and 2.7 Gy, respectively.

### Relationship between radiation dose and coronary intervention

**Fig. 3:**
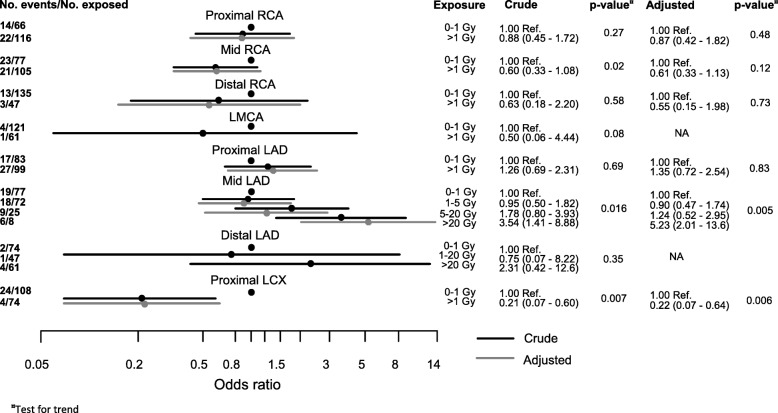
Odds ratio of coronary intervention. Odds ratio (OR) of coronary intervention by coronary artery segment for mean doses. Right coronary artery (RCA), left main coronary artery (LMCA), left anterior descending artery (LAD), left circumflex artery (LCX), confidence interval (CI), Gray (Gy), reference (Ref.), and non applicable (NA). *Adjusted for age at breast cancer (BC) diagnosis, year of BC diagnosis, year of registration in the Swedish Coronary Angiography and Angioplasty Registry (SCAAR), endocrine therapy, chemotherapy, BMI, and smoking status

The ORs of coronary intervention by each coronary segment, for mean and maximum doses were estimated (Fig. 3 and Additional file 1: Table S4). In the mid LAD, a total of 52 coronary events were registered. The odds of coronary events in mid LAD increased with dose (*p*-value for trend test = 0.005).

Women receiving mean doses of 1–5 Gy in the mid LAD had an adjusted OR of 0.90 (95% CI 0.47–1.74), compared to women receiving 0–1 Gy. Women receiving mean doses of 5–20 Gy in the mid LAD had an adjusted OR of 1.24 (95% CI 0.52–2.95) for a later coronary intervention, compared to women receiving 0–1 Gy. Women receiving mean doses over 20 Gy in mid LAD had an adjusted OR of 5.23 (95% CI 2.01–13.6) for a later coronary intervention in comparison with women receiving 0–1 Gy. In distal LAD, there were few interventions and no statistically significant OR for a coronary intervention was found. In the proximal LCX, an adjusted OR of 0.22 (95% CI 0.07–0.64) for a later coronary intervention was seen in women receiving mean doses between 1 and 4 Gy, compared to women receiving 0–1 Gy.

The ORs of coronary intervention over time from the RT exposure were estimated (Table 3). The elapsed treatment time of 0–4 years, 4–8 years, and over 8 years was studied, with elapsed treatment time of 4–8 years defined as reference. There seemed to be an accumulation of interventions in the first 4 years after RT and at elapsed treatment times of 8 years or longer. When time was combined with dose, an OR of 8.21 (95% CI 2.07–32.5) for mean dose over 15 Gy and of 5.39 (95% CI 1.72–16.9) for maximum dose over 40 Gy was observed for coronary events in mid LAD and elapsed treatment time of 8 years or longer. Mean doses of 0–5 Gy, maximum doses of 0–10 Gy, and elapsed treatment time of 0–4 years were defined as reference (Additional file 1: Table S5).

**Table 3:** Odds ratio of coronary event over time

	Number of women	Number of events	Crude analysis		Adjusted model^a^	
			OR	95% CI	OR	95% CI
Proximal RCA						
0–4 years	27	7	2.31	(0.98–5.44)	2.22	(0.94–5.23)
4–8 years	49	21	1.00	Ref.	1.00	Ref.
8+ years	106	8	1.12	(0.41–3.10)	1.18	(0.42–3.29)
Mid RCA						
0–4 years	30	25	1.89	(0.91–3.94)	1.74	(0.83–3.64)
4–8 years	50	10	1.00	Ref.	1.00	Ref.
8+ years	102	9	0.91	(0.37–2.23)	1.00	(0.4–2.48)
Distal RCA						
0–4 years	15	9	2.42	(0.66–8.95)	2.03	(0.54–7.59)
4–8 years	50	3	1.00	Ref.	1.00	Ref.
8+ years	117	4	1.31	(0.29–5.84)	1.71	(0.38–7.82)
LMCA						
0–4 years	8	1	0.82	(0.05–13.1)	NA	
4–8 years	54	1	1.00	Ref.		
8+ years	120	3	2.94	(0.31–28.3)		
Proximal LAD						
0–4 years	24	20	1.20	(0.60–2.42)	1.18	(0.58–2.38)
4–8 years	55	13	1.00	Ref.	1.00	Ref.
8+ years	103	11	0.89	(0.40–1.98)	0.93	(0.41–2.10)
Mid LAD						
0–4 years	30	24	1.30	(0.67–2.51)	1.23	(0.63–2.38)
4–8 years	54	14	1.00	Ref.	1.00	Ref.
8+ years	98	14	1.07	(0.51–2.25)	1.16	(0.55–2.46)
Distal LAD						
0–4 years	9	2	1.65	(0.15–18.2)	NA	
4–8 years	52	1	1.00	Ref.		
8+ years	121	4	3.93	(0.44–35.2)		
Proximal LCX						
0–4 years	18	12	2.40	(0.77–7.45)	2.18	(0.70–6.78)
4–8 years	49	4	1.00	Ref.	1.00	Ref.
8+ years	115	12	3.08	(0.99–9.54)	4.12	(1.31–13.0)
Any segment						
0–4 years	79	78	1.21	(0.82–1.79)	1.10	(0.74–1.63)
4–8 years	45	38	1.00	Ref.	1.00	Ref.
8+ years	58	45	2.22	(1.44–3.43)	2.58	(1.65–4.01)

Odds ratio of coronary event over time. *RCA* Right coronary artery, *LMCA* Left main coronary artery, *LAD* Left anterior descending artery, *LCX* Left circumflex artery, *OR* Odds ratio, *CI* Confidence interval, *Ref.* Reference, and *NA* Non applicable

^a^Adjusted for age at breast cancer (BC) diagnosis, year of BC diagnosis, year of registration in the Swedish Coronary Angiography and Angioplasty Registry (SCAAR), endocrine therapy, chemotherapy, BMI, and smoking status

## Discussion

Our main finding was a positive association between mean radiation doses to mid LAD and a later coronary stenosis that required intervention at that specific location, with a statistically significant five-fold increase of OR when comparing mid LAD mean doses over 20 Gy to doses of 0–1 Gy. Since IHD is a long-term side effect of RT, the combination of dose and time was also studied, which gave an even higher OR than dose alone. In the high-dose region of distal LAD, there were few interventions performed due to the narrow lumen of the vessel, rendering it technically unfeasible. Thus, the prerequisites to study risk of stenosis in this segment were not at hand.

Dose reconstructions were performed in the patients’ original planning-CTs and the information from SCAAR contributed with patient-specific location of the stenosis and thus confirmed its clinical significance. This study is to the authors’ knowledge the largest retrospective study where dose reconstruction to the CAs has been performed in the patient’s original planning CT. This enables a reliable estimation of radiation doses to the specific site where the stenosis was subsequently localised, thus taking the patient-specific thoracic anatomy and dose distribution into account. Due to individual variation in anatomy, radiation dose distribution can differ significantly [23].

In the proximal LCX, a statistically significant decreased OR of 0.22 for a later coronary intervention was seen in women receiving mean doses of 1–4 Gy compared to women receiving 0–1 Gy, thus suggesting lower risk of coronary intervention at higher doses. Since several studies have shown an increase in coronary stenosis at higher radiation doses [4, 16], there seem to be no biological rationale supporting higher doses being protective. The threshold radiation dose for injury to the coronary arteries is unknown and it may be that 1–4 Gy in LCX is a radiation dose too low, to affect our endpoint of coronary injury, PCI. The relevance of this finding is unclear, and due to the small number of events at this location and the low radiation doses, it may be a chance finding.

The estimation of radiation doses in the present study still suffers from uncertainties. The CAs move due to cardiac and breathing movements, while the dose estimation is done in a static image. Steep dose gradients in the anatomical region may lead to uncertainty in dose estimations in small structures like the CAs. The slice thickness of the planning-CTs varied due to technical improvements over time, with thicker slices in the beginning of the study period, implicating fewer dose calculation points. The majority of the patients had planning-CTs with a slice thickness of 5 to 10 mm. Since the contouring was adjusted to encompass the CAs with a diameter of 4 to 6 mm, the CT slice thickness of patients planning-CTs is a limitation with the study. This may contribute to uncertainties in dose estimations. Furthermore, all planning-CTs in the present study were performed without intravenous contrast media according to the standard of care in the clinics, and it is likely that using contrast enhancement would have improved the consistency of the contouring of the CAs.

However, a subgroup of 32 women were initially studied to assess the degree of inter-observer variation in delineating the CAs in the patient’s planning-CT and the results have been published elsewhere [12]. To summarise, the RCA, the LMCA, and the LAD were delineated by three radiation oncologists in a blinded procedure, and the contouring was made by hand in the same manner as in the present study. The distances between the centres of the delineated arteries were measured, and to assess the variance in estimated doses, the intraclass correlation coefficient (ICC) was derived as a measure of agreement between the delineating oncologists. A median mean distance of 2 to 8 mm in the RCA, and of 1 to 4 mm in the LMCA-LAD, was seen between the centres of the different delineations. An ICC of 0.77 to 0.91 was seen for RCA and of 0.84 to 0.99 for the LMCA-LAD, which was considered as an acceptable consistency [12].

Another limitation is the use of the PB dose calculation algorithm in 11.5% of the patients, which may influence the dose estimates close to low-density tissues, as the lung. Thorsen et al. have shown when recalculating from PB to AAA dose algorithms, that there are no significant differences between the estimated doses to the LMCA and LAD in left-sided BC, but a significant 0.36–0.42 Gy decrease of the estimated MHD [24]. Moreover, only small differences were observed in a subset of patients in this study when dose estimation to the CAs between the PB and the CC algorithms were compared and thus the use of different algorithms was considered less likely to affect the results.

The dose-response assessments of CA stenosis were based on the comparison of risk of stenosis in segments receiving low doses with segments receiving high doses. Absolute risk cannot be estimated due to lack of a control group of patients receiving RT without developing CA stenosis. Neither was it possible to quantify an estimated increase in coronary events per Gy, due to the relatively low number of events. The distal LAD received the highest estimated doses but had few interventions. PCI is rarely technically feasible in the narrow lumen of distal LAD, which is an explanation to the low intervention and event rate. Since the choice of end-point in the study was coronary event requiring intervention, based on information from SCAAR, it was not possible to consider any radiation-induced damage to parts of the heart supplied by the distal LAD.

The patients in the present study were treated before techniques of ABC were implemented in Sweden. Since the MHD and doses to the LAD are shown to be considerably lower with these techniques compared to conventional RT techniques, the risk of IHD would likely be lower when ABC techniques are used [13, 14].

Whether the dose to the LAD or the MHD is the best predictor of later coronary events has been studied in recent studies [4, 16, 25]. Darby et al. showed an increase in coronary events of 7.4% per Gy in MHD, but prediction was not improved by including LAD in the model [4]. However, in a more recent study from the same group, doses to LAD were shown to be relevant [18]. In a study using modern 3DCRT and delineating the cardiac structures, the dose to the left ventricle (LV-V5) seemed to be a better predictor for acute cardiac events than the mean heart dose [16]. These studies recorded coronary events of any site, not only in LAD. In contrast, a study by Moignier et al. [25] of Hodgkin’s lymphoma patients estimated doses to specific coronary segments and later stenosis at these sites. In agreement with the present study, a significant dose-response relationship between dose and a later coronary stenosis in this segment was shown, with an estimated increase in coronary events of 4.9% per Gy [25]. In a previous study in BC patients by our group, by estimating radiation doses in specific coronary parts, a significant association between doses to individual coronary segments and later stenosis at high-dose regions was found [10]. Thus, even if MHD is a significant risk factor for the overall risk of later coronary event, these three studies demonstrate a significant association between radiation doses to specific coronary artery segments and later stenosis at the same site.

## Conclusions

In women receiving 3DCRT for BC between 1992 and 2012, radiation doses to the LAD remained high and were associated with an increased requirement of coronary intervention in mid LAD. The results support that radiation dose to the LAD should be considered in RT treatment planning and kept as low as possible, while assuring full target coverage. Further development and optimisation of treatment techniques in order to reduce the dose to LAD, and implementation of heart-sparing RT techniques in the clinical practice is of importance, since minimising the dose to LAD is expected to diminish the risk of later radiation-induced stenosis.

## Additional file

Additional file 1: **Figure S1.** Flow chart showing the selection of women included in the study. **Table S1.** Maximum and mean doses shown in percent of the planned dose to the target. **Table S2.** Patients characteristics by laterality of the tumour. **Table S3.** Distribution of maximum and mean radiation dose exposures in all women. **Table S4.** Odds ratio of intervention by coronary artery segment for maximum doses. **Table S5.** Odds ratio of coronary event by time since RT start and estimated mean and maximum radiation doses to the mid LAD. (DOCX 79 kb)

## Abbreviations

3DCRT: Three-dimensional conformal radiotherapy
AAA: Analytical Anisotropic Algorithm
ABC: Active breathing control
BC: Breast cancer
BCS: Breast conserving surgery
BMI: Body mass index
CABG: Coronary artery by-pass grafting
CAs: Coronary arteries
CC: Collapsed Cone dose algorithm
CI: Confidence interval
DVH: Dose-volume histogram
Gy: Gray
ICC: Intraclass correlation coefficient
IHD: Ischemic heart disease
LAD: Left anterior descending artery
LCX: Left circumflex artery
LMCA: Left main coronary artery
MHD: Median heart dose
OR: Odds ratio
PB: Pencil Beam dose algorithm
PCI: Percutaneous coronary intervention
Q1: First quartile
Q3: Third quartile
RCA: Right coronary artery
RT: Radiotherapy
SCAAR: Swedish Coronary Angiography and Angioplasty Registry
TPS: Treatment planning system
