# Supplementary material for: The relationship between radiation doses to coronary arteries and location of coronary stenosis requiring intervention in breast cancer survivors
Source: Radiat Oncol. 2019 Mar 7;14:40. doi: 10.1186/s13014-019-1242-z (PMC6407212; doi:10.1186/s13014-019-1242-z)
Supplement: Supplementary file 1 — Figure S1. Flow chart showing the selection of women included in the study. Table S1. Maximum and mean doses shown in percent of the planned dose to the target. Table S2. Patients characteristics by laterality of the tumour. Table S3. Distribution of maximum and mean radiation dose exposures in all women. Table S4. Odds ratio of intervention by coronary artery segment for maximum doses. Table S5. Odds ratio of coronary event by time since RT start and estimated mean and maximum radiation doses to the mid LAD. (DOCX 79 kb) [file 13014_2019_1242_MOESM1_ESM.docx]

Additional file 1

Figure S1: Flow chart showing the selection of women included in the study.

Women with BC registered in SCAAR

(n=872)

Not receiving adjuvant RT according to the BC Quality of care registers

(n=270)

RT planned in old TPS not in use or

RT plans not being archived longer or

no 3DCRT

(n=312)

Coronary event registered before RT

(n=15)

Included in the study

(n=182)

Previous BC not recorded in the BC Quality of care registers

(n=8)

Women receiving adjuvant RT and registered in SCAAR

(n=602)

No RT given or RT given at clinic outside the three studied health care regions

(n=85)

Abbreviations: Breast cancer (BC), Swedish Coronary Angiography and Angioplasty Registry (SCAAR), radiotherapy (RT), treatment planning system (TPS), and three-dimensional conformal radiotherapy (3DCRT).

Table S1: Maximum and mean doses shown in percent of the planned dose to the target.

| **Patient with left-sided RT** | Max dose CC | Max dose PB | Mean dose CC | Mean dose PB |
| --- | --- | --- | --- | --- |
| Proximal and mid RCA | 4.5 % | 5.5 % | 3.0 % | 4.0 % |
| Mid and distal LAD | 92.5 % | 94.5 % | 37.5 % | 37.5 % |
| Whole Heart | 96.0 % | 99.0 % | 6.5 % | 7.0 % |

| **Patient with right-sided RT** | Max dose CC | Max dose PB | Mean dose CC | Mean dose PB |
| --- | --- | --- | --- | --- |
| Proximal and mid RCA | 3.0 % | 3.5 % | 1.5 % | 2.5 % |
| Mid and distal LAD | 1.0 % | 1.5 % | 1.0 % | 1.0 % |
| Whole Heart | 5.5 % | 4.0 % | 1.0 % | 1.0 % |

Abbreviations: Maximum (max), Collapsed cone algorithm (CC), Pencil beam algorithm (PB), radiotherapy (RT), right coronary artery (RCA), and left anterior descending artery (LAD).

Table S2: Patients characteristics by laterality of the tumour.

|  | **Right-sided BC**  **(*N*=81)** | | **Left-sided BC**  **(*N*=101)** | | **All**  **(*N*=182)** | | **p-value** |
| --- | --- | --- | --- | --- | --- | --- | --- |
| **Health Care Region, *N* (%)** |  |  |  |  |  |  |  |
| Stockholm | 19 | (23.5) | 28 | (27.7) | 47 | (25.8) | 0.33 |
| Uppsala-Örebro | 41 | (50.6) | 40 | (39.6) | 81 | (44.5) |  |
| Northern | 21 | (25.9) | 33 | (32.7) | 54 | (29.7) |  |
| **Mode of detection, *N* (%)** |  |  |  |  |  |  |  |
| Screening detected | 36 | (44.4) | 44 | (43.6) | 80 | (44.0) | 0.23 |
| Not screening detected | 21 | (25.9) | 20 | (19.8) | 41 | (22.5) |  |
| Missing data | 24 | (29.6) | 37 | (36.6) | 61 | (33.5) |  |
| **Cancer type, *N* (%)** |  |  |  |  |  |  |  |
| Invasive | 75 | (92.6) | 91 | (90.1) | 166 | (91.2) | 0.64 |
| In situ | 6 | (7.4) | 8 | (7.9) | 14 | (7.7) |  |
| Missing data | 0 | (0.0) | 2 | (2.0) | 2 | (1.1) |  |
| **Tumour size, *N* (%)** |  |  |  |  |  |  |  |
| ≤20 mm | 61 | (75.3) | 75 | (74.3) | 136 | (74.7) | 0.61 |
| 20.1-50 mm | 15 | (18.5) | 17 | (16.8) | 32 | (17.6) |  |
| >50 mm | 2 | (2.5) | 1 | (1.0) | 3 | (1.6) |  |
| Missing data | 3 | (3.7) | 8 | (7.9) | 11 | (6.0) |  |
| **N-stage, *N* (%)** |  |  |  |  |  |  |  |
| N0 | 55 | (67.9) | 77 | (76.2) | 132 | (72.5) | 0.36 |
| N1 | 15 | (18.5) | 15 | (14.9) | 30 | (16.5) |  |
| N2 | 0 | (0.0) | 1 | (1.0) | 1 | (0.5) |  |
| NX/Missing data | 11 | (13.6) | 8 | (7.9) | 19 | (10.4) |  |
| **Grade, *N* (%)** |  |  |  |  |  |  |  |
| 1 | 13 | (23.6) | 10 | (15.2) | 23 | (19.0) | 0.31 |
| 2 | 26 | (47.3) | 26 | (39.4) | 52 | (43.0) |  |
| 3 | 7 | (12.7) | 13 | (19.7) | 20 | (16.5) |  |
| Missing data | 9 | (16.4) | 17 | (25.8) | 26 | (21.5) |  |
| **ER-status, *N* (%)** |  |  |  |  |  |  |  |
| ER+ | 54 | (66.7) | 66 | (65.3) | 120 | (65.9) | 0.97 |
| ER- | 11 | (13.6) | 15 | (14.9) | 26 | (14.3) |  |
| Missing data | 16 | (19.8) | 20 | (19.8) | 36 | (19.8) |  |
| **PR-status, *N* (%)** |  |  |  |  |  |  |  |
| PR+ | 44 | (54.3) | 48 | (47.5) | 92 | (50.5) | 0.63 |
| PR- | 21 | (25.9) | 28 | (27.7) | 49 | (26.9) |  |
| Missing data | 16 | (19.8) | 25 | (24.8) | 41 | (22.5) |  |
| **BMI at date of SCAAR registration,**  ***N* (%)** |  |  |  |  |  |  |  |
| Underweight (BMI<20) | 2 | (2.5) | 1 | (1.0) | 3 | (1.6) | 0.76 |
| Normal (BMI 20-25) | 29 | (35.8) | 29 | (28.7) | 58 | (31.9) |  |
| Overweight (BMI 25-30) | 24 | (29.6) | 32 | (31.7) | 56 | (30.8) |  |
| Obese (BMI>30) | 14 | (17.3) | 21 | (20.8) | 35 | (19.2) |  |
| Missing data | 12 | (14.8) | 18 | (17.8) | 30 | (16.5) |  |
| **Smoking status at date of SCAAR registration, *N* (%)** |  |  |  |  |  |  |  |
| Never smoker | 35 | (43.2) | 46 | (45.5) | 81 | (44.5) | 0.93 |
| Current/former smoker | 38 | (46.9) | 46 | (45.5) | 84 | (46.2) |  |
| Unknown | 8 | (9.9) | 9 | (8.9) | 17 | (9.3) |  |
| **Cause of registration, *N* (%)** |  |  |  |  |  |  |  |
| PCI | 78 | (96.3) | 94 | (93.1) | 172 | (94.5) | 0.52 |
| Angiography | 3 | (3.7) | 7 | (6.9) | 10 | (5.5) |  |
| **Indication, *N* (%)** |  |  |  |  |  |  |  |
| STEMI | 22 | (27.2) | 28 | (27.7) | 50 | (27.5) | 0.21 |
| NSTEMI | 30 | (37.0) | 25 | (24.8) | 55 | (30.2) |  |
| Stable angina | 15 | (18.5) | 24 | (23.8) | 39 | (21.4) |  |
| Unstable angina | 11 | (13.6) | 13 | (12.9) | 24 | (13.2) |  |
| Non specified | 3 | (3.7) | 11 | (10.9) | 14 | (7.7) |  |
| **Angiographic findings *N* (%)** |  |  |  |  |  |  |  |
| 1-branch stenosis not main artery | 46 | (56.8) | 54 | (53.5) | 100 | (54.9) | 0.44 |
| 2-branches stenosis not main artery | 23 | (28.4) | 22 | (21.8) | 45 | (24.7) |  |
| 3-branches stenosis not main artery | 7 | (8.6) | 11 | (10.9) | 18 | (9.9) |  |
| Main artery stenosis | 2 | (2.5) | 2 | (2.0) | 4 | (2.2) |  |
| Normal / atheromatosis | 2 | (2.5) | 9 | (8.9) | 11 | (6.0) |  |
| Missing data | 1 | (1.2) | 3 | (3.0) | 4 | (2.2) |  |

Abbreviations: Breast cancer (BC), Estrogen receptor (ER), Progesterone receptor (PR), Swedish Coronary Angiography and Angioplasty Registry (SCAAR), body mass index (BMI), percutaneous coronary intervention (PCI), ST elevation myocardial infarction (STEMI), and non-ST elevation myocardial infarction (NSTEMI).

Table S3: Distribution of maximum and mean radiation dose exposures in all women.

|  | **Max dose in segment** | | | | |  | **Mean dose in segment** | | | | |
| --- | --- | --- | --- | --- | --- | --- | --- | --- | --- | --- | --- |
|  | min | Q1 | median | Q3 | max |  | min | Q1 | median | Q3 | max |
|  | All women (*N*=182) | | | | | | | | | | |
| Proximal RCA | 0.0 | 1.2 | 1.5 | 2.1 | 23.2 |  | 0.0 | 0.9 | 1.2 | 1.7 | 9.2 |
| Mid RCA | 0.1 | 1.0 | 1.6 | 2.2 | 27.8 |  | 0.0 | 0.7 | 1.1 | 1.7 | 5.9 |
| Distal RCA | 0.0 | 0.7 | 1.0 | 1.4 | 3.6 |  | 0.0 | 0.5 | 0.7 | 1.0 | 2.3 |
| LMCA | 0.0 | 0.7 | 1.0 | 1.4 | 19.8 |  | 0.0 | 0.6 | 0.8 | 1.1 | 18.4 |
| Proximal LAD | 0.0 | 0.6 | 1.3 | 2.0 | 26.4 |  | 0.0 | 0.6 | 1.1 | 1.6 | 17.2 |
| Mid LAD | 0.0 | 0.6 | 3.3 | 10.7 | 51.0 |  | 0.0 | 0.5 | 2.1 | 3.7 | 39.6 |
| Distal LAD | 0.0 | 0.7 | 5.7 | 45.6 | 56.7 |  | 0.0 | 0.4 | 3.4 | 30.2 | 52.6 |
| Proximal LCX | 0.0 | 0.6 | 1.1 | 1.4 | 19.6 |  | 0.0 | 0.4 | 0.9 | 1.3 | 9.6 |
| Heart | 0.7 | 3.8 | 40.4 | 47.9 | 57.5 |  | 0.0 | 0.7 | 1.4 | 2.8 | 10.0 |
|  | Right-sided breast cancer (*N*=81) | | | | | | | | | | |
| Proximal RCA | 0.1 | 1.3 | 1.7 | 2.2 | 4.7 |  | 0.1 | 0.8 | 1.3 | 1.7 | 3.5 |
| Mid RCA | 0.1 | 1.7 | 2.2 | 2.7 | 27.8 |  | 0.1 | 1.3 | 1.6 | 2.0 | 5.9 |
| Distal RCA | 0.1 | 1.0 | 1.3 | 1.7 | 3.6 |  | 0.0 | 0.6 | 0.9 | 1.2 | 2.3 |
| LMCA | 0.0 | 0.4 | 0.7 | 1.0 | 16.3 |  | 0.0 | 0.4 | 0.6 | 0.9 | 4.1 |
| Proximal LAD | 0.0 | 0.3 | 0.6 | 0.9 | 2.2 |  | 0.0 | 0.2 | 0.5 | 0.8 | 2.1 |
| Mid LAD | 0.0 | 0.2 | 0.5 | 0.9 | 2.5 |  | 0.0 | 0.1 | 0.5 | 0.8 | 2.3 |
| Distal LAD | 0.0 | 0.1 | 0.5 | 0.9 | 2.5 |  | 0.0 | 0.1 | 0.3 | 0.8 | 2.3 |
| Proximal LCX | 0.0 | 0.3 | 0.6 | 0.8 | 2.0 |  | 0.0 | 0.2 | 0.4 | 0.7 | 1.8 |
| Heart | 0.7 | 2.9 | 3.5 | 5.2 | 41.5 |  | 0.0 | 0.4 | 0.6 | 1.0 | 2.3 |
|  | Left-sided breast cancer (*N*=101) | | | | | | | | | | |
| Proximal RCA | 0.0 | 1.2 | 1.5 | 1.9 | 23.2 |  | 0.0 | 1.0 | 1.1 | 1.7 | 9.2 |
| Mid RCA | 0.1 | 0.8 | 1.1 | 1.5 | 6.7 |  | 0.0 | 0.6 | 0.8 | 1.1 | 3.3 |
| Distal RCA | 0.0 | 0.5 | 0.8 | 1.1 | 2.8 |  | 0.0 | 0.4 | 0.6 | 0.9 | 2.3 |
| LMCA | 0.0 | 1.0 | 1.2 | 1.6 | 19.8 |  | 0.0 | 0.8 | 1.0 | 1.3 | 18.4 |
| Proximal LAD | 0.1 | 1.6 | 2.0 | 2.4 | 26.4 |  | 0.1 | 1.2 | 1.5 | 1.9 | 17.2 |
| Mid LAD | 0.2 | 4.7 | 7.9 | 34.6 | 51.0 |  | 0.1 | 2.4 | 3.6 | 6.2 | 39.6 |
| Distal LAD | 1.8 | 27.9 | 44.8 | 48.1 | 56.7 |  | 1.5 | 7.0 | 26.7 | 41.0 | 52.6 |
| Proximal LCX | 0.0 | 1.1 | 1.4 | 1.8 | 19.6 |  | 0.0 | 0.9 | 1.2 | 1.5 | 9.6 |
| Heart | 1.8 | 45.0 | 47.3 | 49.4 | 57.5 |  | 0.1 | 1.7 | 2.7 | 4.2 | 10.0 |

Distribution of maximum (max) and mean radiation dose exposures in all women, presented for each coronary segment, summarised in descriptive statistics using minimum, first quartile (Q1), median, third quartile (Q3), and maximum. Abbreviations: Right coronary artery (RCA), left main coronary artery (LMCA), left anterior descending artery (LAD), and left circumflex artery (LCX).

Table S4: Odds ratio of intervention by coronary artery segment for maximum doses.

| **Maximum**  **dose** | **Number of**  **women** | **Number of**  **events** | **Crude analysis** | | **Adjusted model*** | |
| --- | --- | --- | --- | --- | --- | --- |
|  |  |  |  | |  | |
| **Proximal RCA** | |  | **OR** | **95% CI** | **OR** | **95% CI** |
| 0-2 Gy | 129 | 25 | 1.00 | Ref. | 1.00 | Ref. |
| >2 Gy^#^ | 53 | 11 | 1.00 | (0.49 - 2.03) | 1.19 | (0.56 - 2.57) |
| **Mid RCA** | |  |  |  |  |  |
| 0-2 Gy | 124 | 36 | 1.00 | Ref. | 1.00 | Ref. |
| 2-5 Gy^#^ | 58 | 8 | 0.44 | (0.20 - 0.95) | 0.50 | (0.22 - 1.12) |
| **Distal RCA** | |  |  |  |  |  |
| 0-2 Gy | 163 | 14 | 1.00 | Ref. | 1.00 | Ref. |
| 2-5 Gy^#^ | 19 | 2 | 1.06 | (0.24 - 4.68) | 1.34 | (0.27 - 6.59) |
| **LMCA** | |  |  |  |  |  |
| 0-2 Gy | 169 | 4 | 1.00 | Ref. | NA | |
| 2-5 Gy^&^ | 13 | 1 | 3.12 | (0.35 - 27.9) |  |  |
| **Proximal LAD** | |  |  |  |  |  |
| 0-2 Gy | 139 | 33 | 1.00 | Ref. | 1.00 | Ref. |
| 2-5 Gy^&^ | 43 | 11 | 1.05 | (0.53 - 2.08) | 1.19 | (0.58 - 2.47) |
| **Mid LAD** | |  |  |  |  |  |
| 0-2 Gy | 81 | 19 | 1.00 | Ref. | 1.00 | Ref. |
| 2-10 Gy | 53 | 13 | 1.00 | (0.50 - 2.03) | 0.95 | (0.46 - 1.96) |
| 10-40 Gy | 28 | 9 | 1.67 | (0.76 - 3.70) | 1.43 | (0.63 - 3.22) |
| 40-51 Gy | 20 | 11 | 2.49 | (1.18 - 5.23) | 2.48 | (1.07 - 5.74) |
| **Distal LAD** | |  |  |  |  |  |
| 0-2 Gy | 81 | 2 | 1.00 | Ref. | NA | |
| 2-40 Gy | 33 | 1 | 1.28 | (0.12 - 14.13) |  |  |
| 40- 57 Gy | 68 | 4 | 2.31 | (0.42 - 12.60) |  |  |
| **Proximal LCX** | |  |  |  |  |  |
| 0-2 Gy | 162 | 28 | NA | | NA | |
| 2-5 Gy^#^ | 20 | 0 |  |  |  |  |

Abbreviations: Odds ratio (OR), right coronary artery (RCA), left main coronary artery (LMCA), left anterior descending artery (LAD), left circumflex artery (LCX), confidence interval (CI), Gray (Gy), reference (Ref.), and non applicable (NA).

*Adjusted for age at breast cancer (BC) diagnosis, year of BC diagnosis, year of registration in the Swedish Coronary Angiography and Angioplasty Registry (SCAAR), endocrine therapy, chemotherapy, BMI, and smoking status. ^#^Less than 2 % of the women had doses above this limit**.** ^&^Less than 3 % of the women had doses above this limit.

Table S5: Odds ratio of coronary event by time since RT start and estimated mean and maximum radiation doses to the mid LAD.

|  | **Time since RT start** | | | | | | | | |
| --- | --- | --- | --- | --- | --- | --- | --- | --- | --- |
|  | **0-4 years** | | | **4-8 years** | | |  | **8+ years** | |
| **Mean dose** | **No. of events** | **OR** | **95 % CI** | **No. of events** | **OR** | **95 % CI** | **No. of events** | **OR** | **95 % CI** |
| **0-5 Gy** | 15 | 1.00 | Ref. | 13 | 1.16 | (0.55 -2.45) | 9 | 0.90 | (0.39 -2.08) |
| **5-15 Gy** | 5 | 2.03 | (0.71-5.78) | 1 | 0.60 | (0.08 - 4.65) | 2 | 1.48 | (0.31 - 6.95) |
| **15+ Gy** | 4 | 5.33 | (1.71 -16.6) | 0 | 0.00 | (0.00 - ∞) | 3 | 8.21 | (2.07 - 32.5) |
|  |  |  |  |  |  |  |  |  |  |
| **Max dose** | **No. of events** | **OR** | **95 % CI** | **No. of events** | **OR** | **95 % CI** | **No. of events** | **OR** | **95 % CI** |
| **0-10 Gy** | 13 | 1.00 | Ref. | 12 | 1.26 | (0.57 - 2.77) | 7 | 0.8 | (0.32 - 2.04) |
| **10-40 Gy** | 6 | 2.36 | (0.88 -6.37) | 1 | 0.54 | (0.07 - 4.16) | 2 | 1.49 | (0.32 - 6.88) |
| **40+ Gy** | 5 | 2.87 | (0.98 -8.42) | 1 | 0.86 | (0.11- 6.85) | 5 | 5.39 | (1.72 - 16.9) |

Abbreviations: Odds ratio (OR), left anterior descending artery (LAD), radiotherapy (RT), Gray (Gy), number (No.), reference (Ref.), maximum (max) and confidence interval (CI).
